# Design and testing of low intensity laser biostimulator

**DOI:** 10.1186/1475-925X-4-5

**Published:** 2005-01-13

**Authors:** Emil S Valchinov, Nicolas E Pallikarakis

**Affiliations:** 1 Department of Medical Physics, University of Patras, Patras 26500, Greece

## Abstract

**Background:**

The non-invasive nature of laser biostimulation has made lasers an attractive alternative in Medical Acupuncture at the last 25 years. However, there is still an uncertainty as to whether they work or their effect is just placebo. Although a plethora of scientific papers published about the topic showing positive clinical results, there is still a lack of objective scientific proofs about the biostimulation effect of lasers in Medical Acupuncture. The objective of this work was to design and build a low cost portable laser device for stimulation of acupuncture points, considered here as small localized biosources (SLB), without stimulating any sensory nerves via shock or heat and to find out a suitable method for objectively evaluating its stimulating effect. The design is aimed for studying SLB potentials provoked by laser stimulus, in search for objective proofs of the biostimulation effect of lasers used in Medical Acupuncture.

**Methods:**

The proposed biostimulator features two operational modes: program mode and stimulation mode and two output polarization modes: linearly and circularly polarized laser emission. In program mode, different user-defined stimulation protocols can be created and memorized. The laser output can be either continuous or pulse modulated. Each stimulation session consists of a pre-defined number of successive continuous or square pulse modulated sequences of laser emission. The variable parameters of the laser output are: average output power, pulse width, pulse period, and continuous or pulsed sequence duration and repetition period. In stimulation mode the stimulus is automatically applied according to the pre-programmed protocol. The laser source is 30 mW AlGaInP laser diode with an emission wavelength of 685 nm, driven by a highly integrated driver. The optical system designed for beam collimation and polarization change uses single collimating lens with large numerical aperture, linear polarizer and a quarter-wave retardation plate. The proposed method for testing the device efficiency employs a biofeedback from the subject by recording the biopotentials evoked by the laser stimulus at related distant SLB sites. Therefore measuring of SLB biopotentials caused by the stimulus would indicate that a biopotential has been evoked at the irradiated site and has propagated to the measurement sites, rather than being caused by local changes of the electrical skin conductivity.

**Results:**

A prototype device was built according to the proposed design using relatively inexpensive and commercially available components. The laser output can be pulse modulated from 0.1 to 1000 Hz with a duty factor from 10 to 90 %. The average output power density can be adjusted in the range 24 – 480 mW/cm2, where the total irradiation is limited to 2 Joule per stimulation session. The device is controlled by an 8-bit RISC Flash microcontroller with internal RAM and EEPROM memory, which allows for a wide range of different stimulation protocols to be implemented and memorized. The integrated laser diode driver with its onboard light power control loop provides safe and consistent laser modulation. The prototype was tested on the right Tri-Heater (TH) acupuncture meridian according to the proposed method. Laser evoked potentials were recorded from most of the easily accessible SLB along the meridian under study. They appear like periodical spikes with a repetition rate from 0.05 to 10 Hz and amplitude range 0.1 – 1 mV.

**Conclusion:**

The prototype's specifications were found to be better or comparable to those of other existing devices. It features low component count, small size and low power consumption. Because of the low power levels used the possibility of sensory nerve stimulation via the phenomenon of shock or heat is excluded. Thus senseless optical stimulation is achieved. The optical system presented offers simple and cost effective way for beam collimation and polarization change. The novel method proposed for testing the device efficiency allows for objectively recording of SLB potentials evoked by laser stimulus. Based on the biopotential records obtained with this method, a scientifically based conclusion can be drawn about the effectiveness of the commercially available devices for low-level laser therapy used in Medical Acupuncture. The prototype tests showed that with the biostimulator presented, SLB could be effectively stimulated at low power levels. However more studies are needed to derive a general conclusion about the SLB biostimulation mechanism of lasers and their most effective power and optical settings.

## Background

Nowadays lasers are widely used in therapy and diagnostics. They have been adapted to many medical procedures ranging from surgery, oncology, physiotherapy, dentistry, dermatology and biostimulation. The non-invasive nature of laser biostimulation have made lasers an attractive alternative in Medical Acupuncture at the last 25 years. Unfortunately, there is still an uncertainty as to whether they work or their effect is just placebo. Although a plethora of scientific papers published about the topic showing positive clinical results, there is still a lack of objective scientific proofs about the biostimulation effect of lasers in Medical Acupuncture.

The properties of acupuncture points, considered here as small localized biosources (SLB), have been extensively studied over the past 50 years. Research has shown SLB to be small area body regions, which exhibit unique, electrical, physiological and anatomical properties (e.g. high density of gap junctions, relatively low impedance etc.). They are considered to form groups, each group being arranged along a line, called meridian and related to an internal organ [1-4]. SLBs appear to be highly sensitive to mechanical, thermal, electrical or electromagnetic stimulation and are found to take place from the epidermis to a maximum depth of 2 cm [5-8]. It has been shown that with proper laser wavelength, intensity and collimation, low-level laser energy could be effectively delivered to SLB up to a 10 mm beneath the skin surface [9].

The objective of this work was to design and build a low cost portable laser device for effectively stimulation of SLB without exciting sensory nerves, and to find out a suitable method for objectively evaluating its efficiency. The attempt to define the optimal device parameters was based on the SLB properties, data about existing devices for low level laser therapy and on preliminary measurements performed in our laboratory. The latter suggest that the effect of SLB stimulation is also dependent on the polarization of the coherent emission in addition to its intensity, wavelength and modulation frequency. Therefore the device should provide a polarization adjustment, wide range of modulation frequencies, precise power settings and to have minimum size and cost.

## Methods

### Basic design and operating principle

**Figure 1:**
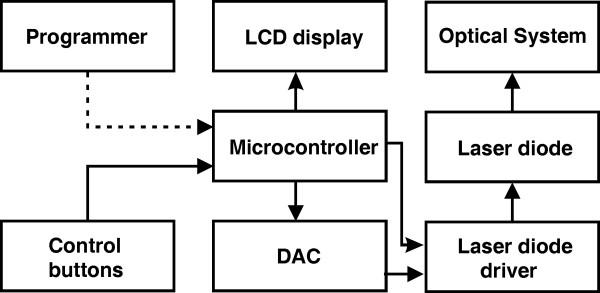
Block diagram of the of the laser biostimulator.

**Figure 2:**
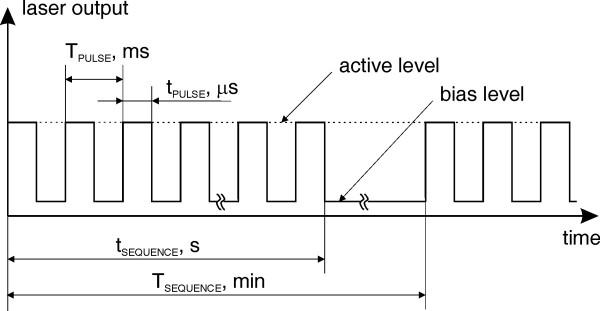
Time diagram of pulse modulated laser output.

The block diagram of the proposed biostimulator design is shown in Fig. 1. A laser diode is used as a coherent source of radiation because of its high brightness, efficiency, low cost and possibility for direct modulation. The emission wavelength is chosen in the visible range for minimum water absorption and haemoglobin reflection. The diode is driven by a microcontroller through an integrated driver and digital-to-analog converter (DAC). The user interface includes a liquid crystal display (LCD) and control buttons. The connection with the programmer is optional and is used only for in-circuit serial programming (ICSP). The optical system serves for beam collimation and polarization adjustment. Each stimulation session contains certain number of consecutive sequences of laser emission, where every sequence consists of continuous or pulse modulated laser emission as shown in Fig. 2. There are two main operational modes: program mode and stimulation mode, and two output polarization modes: linearly and circularly polarized laser emission. In program mode, a set of different user-defined stimulation protocols can be created and memorized according to the requirements of the specific study.

The variable parameters of the laser output are: average output power, pulse width, pulse period, and sequence duration and repetition period. After each input parameter is selected, the total energy that would be delivered at the end of the stimulation session is automatically calculated and displayed. When defining the stimulation protocol, the software program reads the selected parameter value and automatically re-calculates the possible set of the other parameters, so that the user could not select inconsistent values or ones that would result in a total energy delivered that exceeds a certain safety limit. In stimulation mode the laser stimulus is applied according to the pre-programmed protocol.

**Figure 3:**
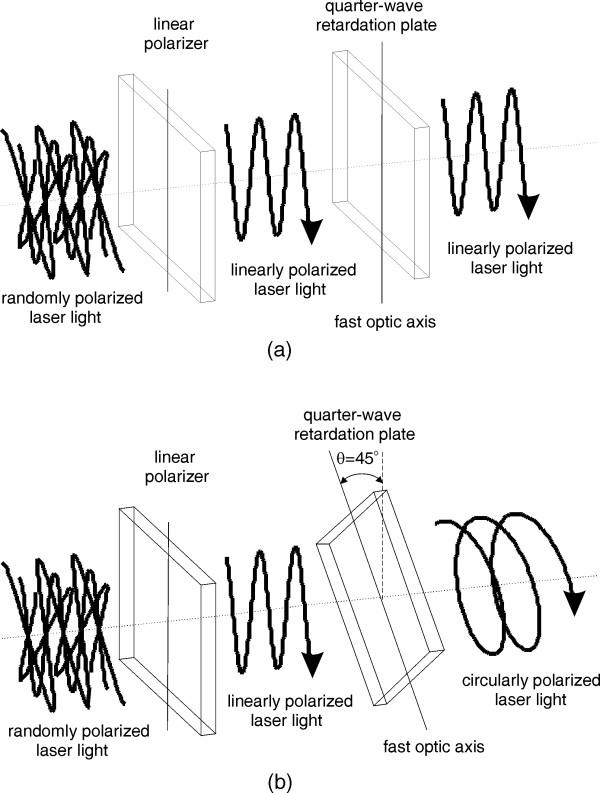
Polarization change principle.

A quarter-wave retardation plate realizes the laser output polarization change, as shown in Fig. 3. Within the retarder plane, the crystalline optic axis and the axis normal to it are also called fast or slow axis, depending on whether the uniaxial crystal is positive or negative. By rotating the retarder slightly about one of these axes, the retardation amount or the phase shift is varied. If the electric field vector of the incident linearly polarized beam and the quarter-wave retarder principal plane coincide, the emergent beam polarization remains the same as shown in Fig. 3a. If the angle *θ *between the electric field vector of the incident linearly polarized beam and the quarter-wave retarder principal plane is +45 degrees, the emergent beam is circularly polarized as shown in Fig. 3b. Reversing *θ *to -45 degrees reverses the sense of the circular polarization. The output from a single cavity laser diode is mainly linearly polarized, parallel to the laser junction. Although, spontaneous emission with a random polarization and with a polarization perpendicular to the laser junction is also present. For a diode operating near its maximum power the polarization ratio is typically greater than 100:1 but when operating near the threshold point, the ratio is considerably lower as spontaneous emission becomes more significant. Therefore a collimating lens followed by a linear polarizer is used since the retarder requires linearly polarized and normally incident light upon its plate over the whole power range. A single collimating lens, with good anti-reflection coating and large numerical aperture to efficiently capture the widely divergent perpendicular axis of the laser diode, is the most efficient and cost effective solution for the current application. Polarizers typically utilize birefringence, dichroism, optical activity, and polarization by reflection or by a metallic thin film [10]. For low-power and visual applications like the current one, sheet-type polarizers utilizing dichroism are normally used. Dichroic sheet polarizers subject one of the two orthogonal polarizations to strong absorption. They offer large apertures and acceptance angles, excellent extinction ratios and are simple to mount.

The application can tolerate an elliptical beam shape and waveform aberrations, so circularization of the laser beam or correction of the waveform aberrations is not required.

### Method for testing the device efficiency

**Figure 4:**
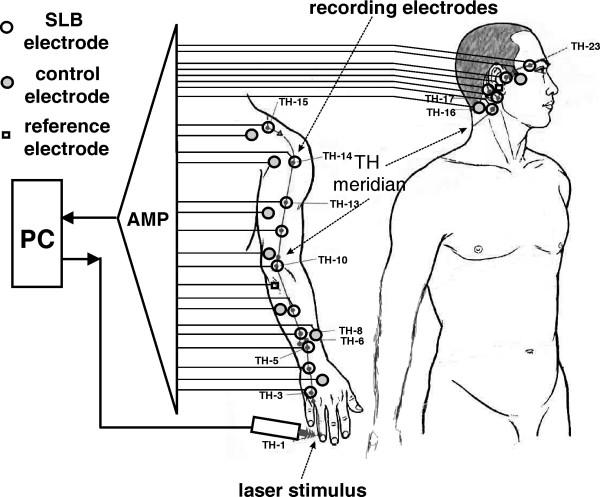
Method for testing the device efficiency.

The best way for testing the device efficiency is to obtain a biofeedback from the site of stimulation. A suitable non-invasive method is to measure the surface biopotential of the irradiated SLB site. However simultaneous stimulation and biopotential recording from a single SLB is technically difficult and inadequate, since the record may contain sham potentials due to local changes of the electrical conductivity of the irradiated skin. So a new method is proposed to avoid this problem. The method uses separate stimulation and measurement sites. Thus the laser stimulus is applied at SLB situated at the beginning of the meridian (e.g. the first point), where biopotential records are obtained from all the other easily accessible SLB lying distantly on the same meridian (see Fig. 4). Therefore measuring of SLB biopotentials caused by the stimulus would indicate that a biopotential has been evoked at the irradiated site and has propagated to the measurement sites, rather than being caused by local changes of the electrical skin conductivity. Extra electrodes have to be placed at non-SLB sites at close proximity to individual or group of closely spaced SLB recording electrodes, as a control. Due to the low intensity of the biostimulator output, even after prolonged irradiation, the subject has no thermal or tactile sensation and remains unaware of the application of the stimulus. Further more, there should be no visual, auditory or tactile cues that may indicate the activation of the laser.

### Practical biostimulator circuit

**Figure 5:**
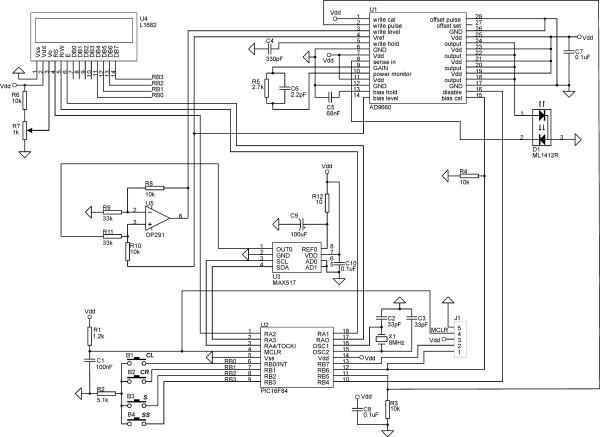
Practical biostimulator circuit.

The schematic of the practical biostimulator circuit, built according to the proposed design is shown in Fig. 5. The microcontroller is implemented with the 8-bit RISC Flash microcontroller PIC16F84 with built in RAM and EEPROM memory [11]. It operates in HS oscillator mode with an 8 MHz crystal resonator. Power-on reset (POR), power-up timer and the oscillator start-up timer are enabled to allow for the power supply to rise to an acceptable level. The input/output ports are configured as follows:

• RA0-RA2 – outputs used as LCD control signals

• RA3-RA4 – outputs, used as DAC control signals

• RB0-RB3 – either inputs or outputs, shared between the input control buttons B1-B4 and the LCD data bus DB4-DB7

• RB4-RB6 – outputs, used as control signals for the laser diode driver

The LCD is implemented with the dot matrix alphanumeric character module U4 (Seiko Instruments L1682). It features low power consumption, high contrast, wide viewing angle, on-board controller and LSI driver (Samsung S6A0069). All functions required for the LCD drive are internally provided on the chip. Its internal operation is determined by signals sent from the microcontroller. These signals include:

• Register select – RS

• Read/Write – R/W

• Data bus – DB4-DB7 (configured as inputs)

• Read/Write Enable – E

When ports RB0-RB3 are configured as inputs, the LCD data inputs DB4-DB7 have no practical influence on the logic levels set by push buttons B1-B4. LCD operation is also not affected since DB4-DB7 content is read only on logic high at E (U4-pin 6), set by the microcontroller [12]. When ports RB0-RB3 are configured as outputs, input buttons B1-B4 cannot alter their output logic levels because of resistor R2 connecting B1-B4 to common. Connector J1 is used for the microcontroller ICSP.

The laser diode driver is implemented with the highly integrated circuit U1 (Analog Devices AD9660), which combines a very fast output current switch with onboard analog light power control loops. It gets feedback current from the laser diode built-in photo detector (U1-pin 8), feeds it to a transimpedance amplifier (TZA) and then to two analog feedback loops where the *bias *and the *active *power levels of the laser are set [13]. The two levels are proportional to the analog input voltage at the *bias *level input (U1-pin 14) and at the *active *level input (U1-pin 3). These inputs drive track and hold amplifiers with hold capacitors C4 and C5. The input voltage range on both inputs ranges from V_ref _to V_ref _+ 1.6 V, requiring an offset of V_ref _to be created for common based signals. The *bias *level is chosen to be equal to V_ref_, where the *active *level is determined by the circuit realized with op-amp U5. It performs the level shift and scales the DAC output from V_ref _to V_ref _+ 1.6 V. This solution is attractive because both DAC and op-amp can run off a single 5 V supply, and the op-amp does not have to swing rail-to-rail. The op-amp U5 output voltage level is given by:



Since the monitor current is proportional to the laser diode light power, the feedback loops effectively control the laser power to a level proportional to the analog inputs. The *bias *control loops is periodically calibrated via U1-pin 15, where the *active *control loop is continuously calibrated via U1-pin 1. Resistors R3 and R4 are used to avoid floating of inputs U1-pin 15 and U1-pin 2 when microcontroller ports are in a high impedance mode. The laser pulse modulation is done by switching between the *bias *and the *active *power levels according to the logic level at U1-pin 2, where logic high turns the modulation current on. The gain resistor R5 matches the feedback loop transfer function to the laser/photo diode D1. Capacitor C6 optimizes the TZA response, with larger values to slow TZA response. Lower values increase TZA bandwidth but may cause oscillations. When input U1-pin 16 is logic high, the onboard disable circuit turns off the output drivers and returns the light power control loops to a safe state. It is used during initial power up of U1 and when the laser is inactive. In case that input U1-pin 16 floats (after POR or other reset conditions) the driver is disabled. When U1 is re-enabled the control loops are recalibrated.

The DAC is implemented with the single 8-bit voltage output MAX517 (U3). It is controlled by the microcontroller via 2-wire serial interface (U3-pin 3, U3-pin 4), operates from a single power supply and swings rail-to-rail. POR ensures the DAC output is at zero volts when power is initially applied. It uses the power supply V_dd _as reference (U3-pin 8) filtered by R12 and C9. The DAC's full-scale output voltage ranges from 0 to V_dd_. Special attention was paid to the PCB layout design to minimize the crosstalk between analog inputs and digital outputs.

**Figure 6:**
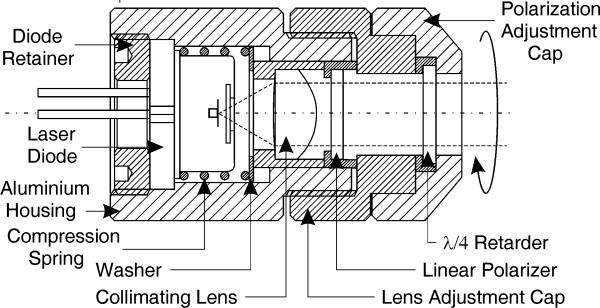
Optical system assembly.

The drawing of the practical optical system assembly is shown in Fig. 6. It includes an aluminium housing, collimating lens, linear polarizer and a quarter-wave retarder. Turning manually the polarization adjustment cap, clockwise or anti-clockwise at 45°, changes the polarization from linear to circular left or right-handed. The laser source used is a high power AlGaInP (Mitsubishi ML1412R) laser diode, which provides a stable, single transverse mode oscillation with a typical emission wavelength of 685 nm and a maximum continuous output power of 30 mW. The diode is matched with a single collimating lens with a relatively large numerical aperture (NA) and a single layer of anti-reflection coating (MgF_2_), optimized for 670 nm. The polarizer used is a linear polarizing film produced by aligning long chain polymers, which is then laminated in cellulose acetate butyrate (CAB) for durability and stiffness [14]. The quarter-wave retarder is implemented with polyvinyl-alcohol film supported by CAB.

## Results

A prototype device was built according to the proposed design using inexpensive and commercially available components. Its optical and electrical characteristics are given in Table 1. The microcontroller's internal RAM and EEPROM memory allows for a wide range of different stimulation protocols to be implemented and memorized. The integrated laser diode with its onboard light power control loop provides safe and consistent laser modulation. Because the application of the stimulus throughout the stimulation session is entirely controlled by the microcontroller, once the procedure is started, both subject and operator are unaware of whether the stimulus is active or not, except if intentionally staring the stimulated location. Thus true double-blind studies can be performed. The optical system presented, offers simple and cost effective way for beam collimation and polarization change. With the power levels used the possibility of sensory nerve stimulation via shock or heat is excluded.

**Table 1:** Device specifications.

**Parameter**	**Value**	
Beam dimensions (-3dB)	D_┴_	D_\\_
	3.12 mm	1.7 mm
Average output power	1–20 mW	
Average output power density	24–480 mW/cm^2^	
Maximum irradiation per stimulation session	2 Joule	
Collimating lens coupling efficiency (685 nm, f = 4.6 mm, NA = 0.53)	96 %	
Polarizer transmittance (685 nm, optic axis parallel to the diode junction)	75 %	
Quarter-wave retarder transmittance, (685 nm)	93 %	
Total optical system transmittance (685 nm)	67 %	
Output polarization	linear/circular	
Laser class	IIIb	
Pulse modulation	0–10 000 Hz	
Duty factor	10–90 %	
Pulse sequence duration	1–30 sec	
Pulse sequence repetition period	1–5 min	
Number of pulse sequences	1–20	
Maximum power consumption (5 V)	600 mW	

**Figure 7:**
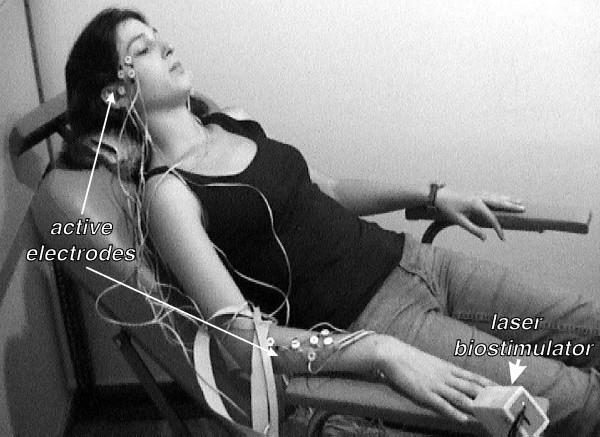
Photo of the electrodes placement and stimulus application during a preliminary measurement.

**Figure 8:**
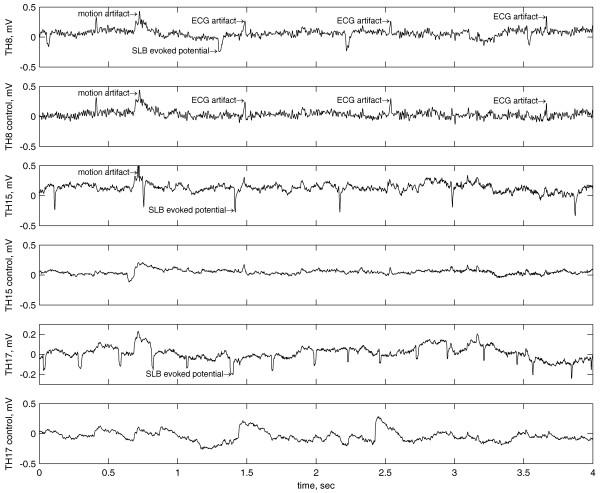
Evoked biopotentials acquired from points TH-8, TH-15, and TH-17 and their controls.

The biostimulator was tested on the right Tri-Heater (TH) meridian of ten subjects according to the proposed method (see Fig. 4). The laser stimulus was applied at point TH-1, where recording electrodes were placed along the same meridian on points TH-3, 4, 5, 6, 7, 8, 9, 13, 15, 16, 17, 18, 21, 22 and 23, which were relatively easily accessible (see Fig. 7). Additional control electrodes were placed at non-SLB sites, approximately 2 cm apart from each SLB electrode, and the reference was positioned at the right ear lobe. Sample records of unprocessed real time SLB laser evoked potentials obtained from points TH-8, TH-15, TH-17 and their controls are shown in Fig. 8. These signals were recorded with an active electrode amplifier [15] from one subject and are responses to the same stimulation, where the biostimulator settings used are given in Table 2. The SLB evoked potentials appear like periodical spikes with a repetition rate from 0.05 to 10 Hz and amplitude range 0.1 – 1 mV. The preliminary results suggest that the repetition rate of the evoked SLB signals is proportional to the total energy delivered by the stimulus.

**Table 2:** Device settings used during the preliminary measurements.

**Parameter**	**Value**
Average output power	10 mW
Average output power density	240 mW/cm^2^
Pulse modulation	2000 Hz
Duty factor	50 %
Pulse sequence duration	10 sec
Pulse sequence repetition period	1 min
Number of pulse sequences	10
Output polarization	Circular

The noise present in the signals is mainly composed of electromyographic signals and noise from the electrode-skin interface. The first two signal records (TH-8 and control) contain ECG artifacts and additional electromyographic noise since those two electrodes were positioned relatively distant from the reference electrode. The frequency bandwidth was limited to 200 Hz by sixth order low-pass Bessel filter and the signals were sampled with 1 kHz.

Recording to the EU regulation (Medical Device Directive 93/94) this device falls under class IIb in order to obtain the CE mark. The biostimulator prototype was categorized as Class IIIb laser product according to the International Standard for the Safety of Medical Laser Products IEC 601-2-22, as stated in Table 1.

## Discussion

The best solution for building a compact hand held biostimulator would be to design a custom made integrated circuit, but the cost would be much higher. We found a good alternative in using surface mount technology (SMT), commercially available integrated laser diode driver and a RISC Flash microcontroller. This solution resulted in a reduction in parts, size and power consumption. The proposed method for testing the device efficiency is very sensitive to precise electrode and stimulus positioning. Even a deviation of 3 mm from the exact SLB location may prevent the recording electrode from capturing signals from the source. The same deviation of the stimulus position also results of ineffective excitation of the targeted SLB and thus no SLB evoked potentials can be recorded. The method is also susceptible to the electrode-skin pressure, but not only due to its strong influence on the contact impedance. It was observed that the excessive electrode-skin pressure led to diminishing or even disappearing of the SLB signal, although the contact impedance was lower. This is most probably due to the pressure exerted on the SLB source that may affect the signal generation or transduction. Alternatively insufficient electrode-skin pressure led to excessive contact impedance and noise from the electrode-skin interface. The preliminary results suggest that a circularly polarized laser emission is most effective when used on the so-called Yang acupuncture meridians but not on Yin types. However more studies are needed to validate or disprove this observation.

## Conclusions

The specifications of the prototype, built according to the proposed design, were found to be better or comparable to those of other existing devices. It features small size and low component count and power consumption. Because of the low power levels used the possibility of sensory nerve stimulation via the phenomenon of shock or heat is excluded. Thus senseless optical stimulation is achieved. The optical system presented offers simple and cost effective way for beam collimation and polarization change. The novel method proposed for testing the device efficiency allows for objectively recording of SLB potentials evoked by laser stimulus. Based on the biopotential records obtained with this method, a scientifically based conclusion can be drawn about the effectiveness of the commercially available devices for low level laser therapy used in Medical Acupuncture. The prototype tests showed that with the biostimulator presented, SLB could be effectively stimulated at low power levels. However more studies are needed to derive a general conclusion about the biostimulation mechanism of lasers in Medical Acupuncture and their most effective power and optical settings.

## Authors' contributions

The authors contributed equally to this work
